# Norcantharidin Facilitates LPS-Mediated Immune Responses by Up-Regulation of AKT/NF-κB Signaling in Macrophages

**DOI:** 10.1371/journal.pone.0044956

**Published:** 2012-09-11

**Authors:** Qufei Zhao, Yu Qian, Ruimei Li, Binghe Tan, Honghui Han, Mingyao Liu, Min Qian, Bing Du

**Affiliations:** Shanghai Key Laboratory of Regulatory Biology, Institute of Biomedical Sciences and School of Life Sciences, East China Normal University, Shanghai, China; Chang Gung University, Taiwan

## Abstract

Norcantharidin (NCTD), a demethylated analog of cantharidin, is a common used clinical drug to inhibit proliferation and metastasis of cancer cells. But the role of NCTD in modulating immune responses remains unknown. Here, we investigated the function and mechanism of NCTD in regulation of TLR4 associated immune response in macrophages. We evaluated the influence of NCTD on host defense against invaded pathogens by acute peritonitis mouse model, ELISA, Q-PCR, nitrite quantification, phagocytosis assay and gelatin zymography assay. Our data showed that the survival and the serum concentrations of IL-6 and TNF-α were all enhanced by NCTD significantly in peritonitis mouse model. Accordingly, LPS-induced cytokine, nitric oxide and MMP-9 production as well as the phagocytosis of bacteria were all up-regulated by NCTD in a dose dependent manner in both RAW264.7 cells and bone marrow-derived macrophages (BMMs). Then we further analyzed TLR4 associated signaling pathway by Western blot, Immunofluorescence and EMSA in the presence or absence of LPS. The phosphorylation of AKT and p65 at serine 536 but not serine 468 was enhanced obviously by NCTD in a dose dependent manner, whereas the degradation of IκBα was little effected. Consequently, the nuclear translocation and DNA binding ability of NF-κB was also increased by NCTD obviously in RAW264.7 cells. Our results demonstrated that NCTD could facilitate LPS-mediated immune response through promoting the phosphorylation of AKT/p65 and transcriptional activity of NF-κB, thus reprofiling the traditional anti-tumor drug NCTD as a novel immune regulator in promoting host defense against bacterial infection.

## Introduction

Innate immune system serves as the first defense to protect the host from invaded pathogens in a non-specific but instant way [1]. Different pathways have been involved to eliminate the invaded pathogens in innate immune system which are different from acquired immune system, including anatomical barriers, mechanical removal, bacterial antagonism, pattern recognition and phagocytosis [2]. Among them, recognition to pathogen-associated molecular patterns (PAMPs) by germline-encoded pattern-recognition receptors (PRRs) is one of most essential way for host to distinguish the pathogens from self-tissues [3]. PRRs can be divided into two distinct classes based on the cellular functions: endocytic pattern-recognition receptors and signaling pattern-recognition receptors. Endocytic pattern-recognition receptors could be found in many phagocytes and promote the attachment to the microorganisms and facilitate the subsequent engulfment and destruction, whereas signaling pattern-recognition receptors promote the synthesis and secretion of immune regulators or molecules such as cytokines and chemokines that are crucial to regulate innate and adaptive immunity [4]–[7].

Toll-like receptors (TLRs), a family of innate immune receptors, are widely expressed on the cell surface of most immune cells. They work as a primary sensor of invading pathogens, which is essential for eliciting the innate response and regulating adaptive immunity [8]. Thirteen TLRs have been found to specifically recognize different PAMPs, such as TLR4 (LPS), TLR1/2 (peptidoglycan), TLR5 (flagellin), TLR8 (ssRNA) and TLR9 (CpG DNA). The activation of TLRs can stimulate immune cells and cause the secretion of cytokines and chemokine such as tumor necrosis factor-alpha (TNF-α), interleukin-6 (IL-6), GM-CSF and MCP-1 to modulate innate and acquired immune response [9]–[11]. The most important signaling provoked by TLRs is the transcription factor NF-κB (nuclear factor-κB), which regulates the expression of many immune and inflammatory genes [12]. Therefore, the screening of small molecular compounds modulating the NF-κB signaling pathway will be an effective approach in regulation of innate immune responses.

Cantharidin (CTD) is the major active component of blister beetles (one type of Chinese traditional medicine), which could be used as antitumor drug in most cancer cells. But the excessive cytotoxicity of CTD limited its clinical application. Whereas NCTD, a demethylated analog of CTD, possesses less cytotoxicity and could be used for more clinical applications [13]. Nowadays, NCTD has been widely used as an anti-tumor drug to inhibit proliferation and metastasis of several kinds of cancers, such as liver cancer [14], lung cancer [15], colorectal cancer [16], breast cancer [17] and oral cancer [18] in China. In addition, NCTD was also found possessing anti-angiogenesis activity in cancer therapy [19]. Previous reports have shown that NCTD not only decreased metastasis and survival but also reduced the tumor volumes and weights in tumor xenograft model of human gallbladder carcinoma in nude mice [20]. Huang's results indicated that NCTD's treatment could induce G2/M phase arrest and reduce Bcl-2/Bax ratio in MDA-MB-231 cells [17].

**Figure 1:**
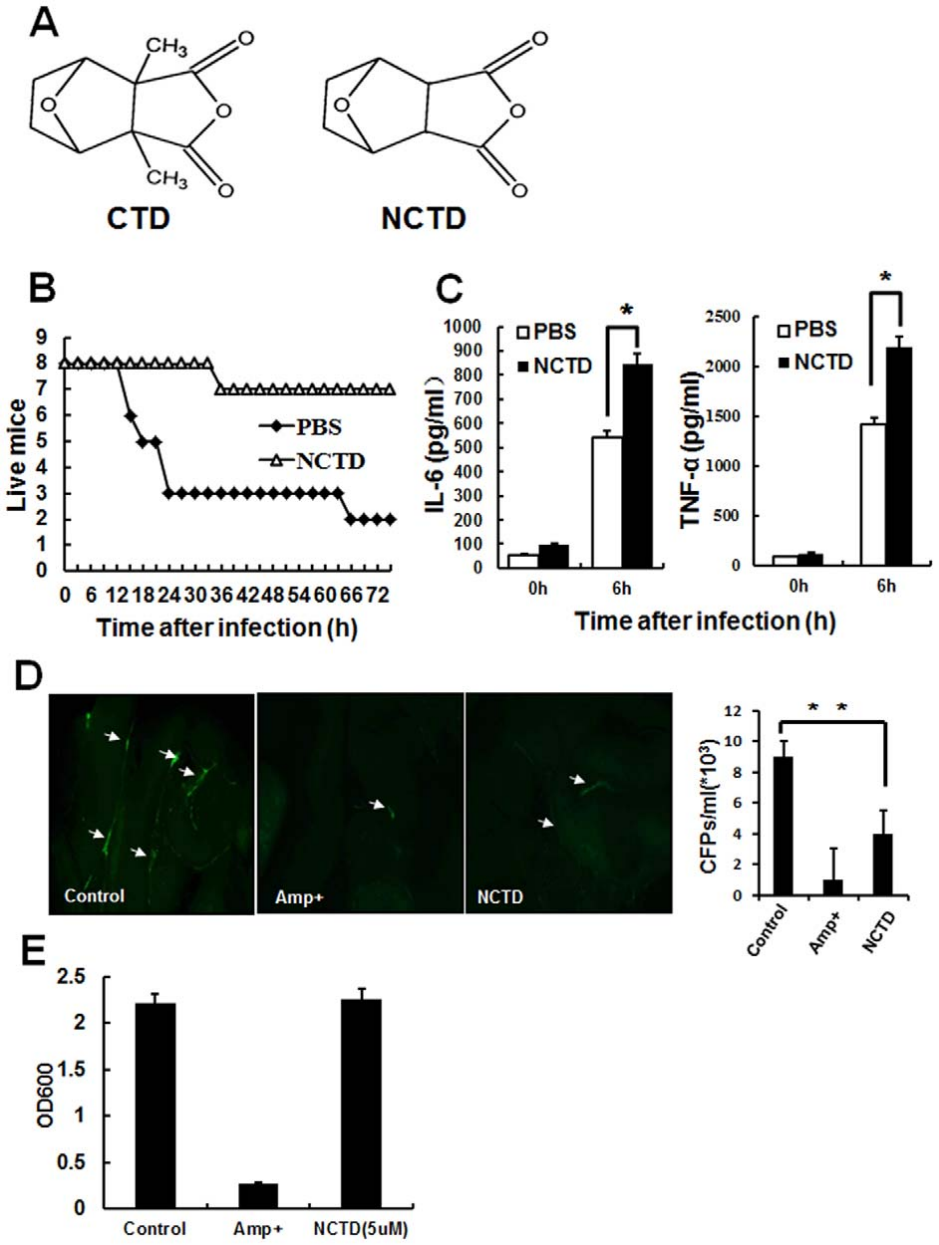
NCTD protects mice from bacterial-induced peritonitis. A, Chemical structure of NCTD and CTD. B, The survival of peritonitis mouse was increased by NCTD obviously. The mice were treated with NCTD or PBS at 24 h and 6 h before infection. Both NCTD and PBS (containing 0.1% DMSO) treated mice (n = 8) were intraperitoneal injected with *E.coli 0111* (8*10^7^) and monitored every 3 h for 72 h. C, Serum concentration of IL-6 and TNF-α in mouse model was enhanced by NCTD. The blood was obtained 6 h after infection and the serum concentration of IL-6 and TNF-α was measured by ELISA assay. (*, P<0.0*5*) Data are representative of three independent experiments with similar results. D, The quantity of bacterial was reduced in NCTD treated mouse. The mice were treated with 1 ml vehicle control (containing 0.1% DMSO), 100 ug/ml Ampicillin or NCTD (10 mg/kg) respectively followed by an i.p injection with 5×10^7^ CFU of *E. coil*-GFP in 1 ml PBS for 16 h. Then the bacteria resident in abdomen was visualized by fluorescent stereomicroscope. Also, their peritoneal cavities were lavaged with 3 ml PBS, and bacterial counts were determined by plating on agar plates. n = 4 mice per group. E, NCTD has no effects on the growth of *E. coil*-GFP. E.coli-GFP were cultured with 100 µg/ml Ampicillin (Amp+) and 5 μM NCTD for 24 h respectively. Then the bacteria number was detected by spectrophotometer. Data are representative of three independent experiments with similar results (mean ± s.d. in B, D).

Here, we provide evidence that NCTD has a non-redundant role in promoting host survival from *E. coli* induced peritonitis. Meanwhile, the LPS-induced cytokines expression and immune responses were enhanced significantly in macrophages by NCTD. Furthermore, the AKT/p65 phosphorylation and NF-κB activities was also enhanced by NCTD in a doses dependent manner. Taken together, NCTD could facilitate LPS-mediated innate immune response significantly through activating AKT/NF-κB signaling, thereby contributing to clearance of invaded pathogens.

**Figure 2:**
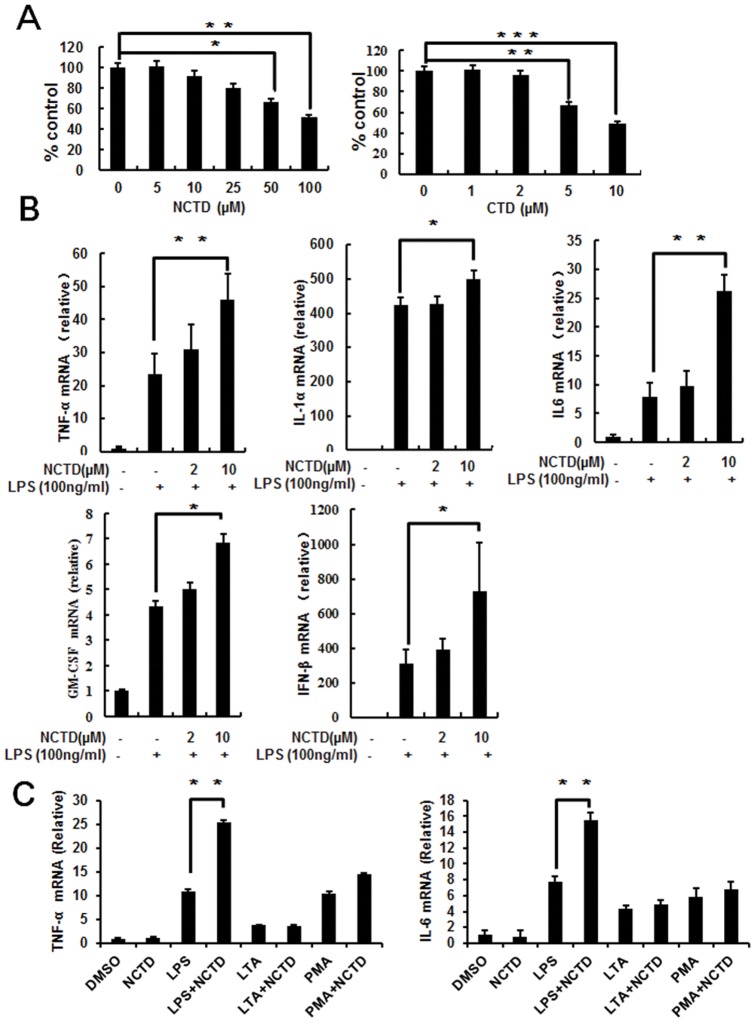
NCTD enhances LPS induced cytokines expression at mRNA level. A, The cytotoxicity of NCTD is much lower than CTD. RAW264.7 cells were treated with NCTD or CTD for 24 h and cell viability was tested with MTS assay as described under Materials and Methods. B, Cytokines production was enhanced obviously by NCTD in RAW264.7 cells. The RAW264.7 cells were pretreated with PBS (containing 0.1% DMSO) or 1–10 μM NCTD for 24 h before stimulation with 100 ng/ml LPS for 1 h. Total cellular RNA were collected and subjected to Real time -PCR analysis. C, Only LPS induced cytokine production was enhance by NCTD obviously. The RAW264.7 cells were pretreated with 0.1% DMSO, 5 μM NCTD for 24 h before stimulation with LPS (100 ng/ml), PMA (100nM) or LTA (10 µg/ml) for 1 h. Total cellular RNA were collected and subjected to Real time PCR analysis. Columns, mean from three independent experiments with three duplicates; bars, SE (*, P<0.05; **, P<0.01 versus control).

## Materials and Methods

### Cells, reagents and animals

NCTD, CTD and LPS were purchased from Sigma. PI3K/AKT inhibitor (20 μM, LY294002) was purchased from Biotime Company (Shanghai, China). NCTD/CTD was prepared in DMSO at 10 mM and stored at −20°C in the dark, then diluted as needed in cell culture medium. RAW264.7 and L929 cells were purchased from China Type Culture Collection (Shanghai, China). RAW264.7 cells were cultured in DMEM supplemented with 5% fetal bovine serum (FBS; Hyclone Laboratories). All the cells were raised at 37°C under a humidified 95/5 (v/v) mixture of air and CO_2_. The female 4–6 weeks C57BL/6 mice were purchased from Bikai Animal Inc. (Shanghai, China) and housed in standard plastic cages under automatic 12 h light:dark cycles at 23°C with free access to food and water. All animals were kept under specific pathogen free conditions and maintained in accordance with institutional guidelines. For peritoneal macrophage isolation, thioglycolate was injected into female mice peritoneum to elicit the macrophages for 3–4 days, and then PBS was used to harvest peritoneal macrophage. Bone marrow-derived macrophages were isolated from 4–6 weeks C57BL/6 mice as described [21].

**Figure 3:**
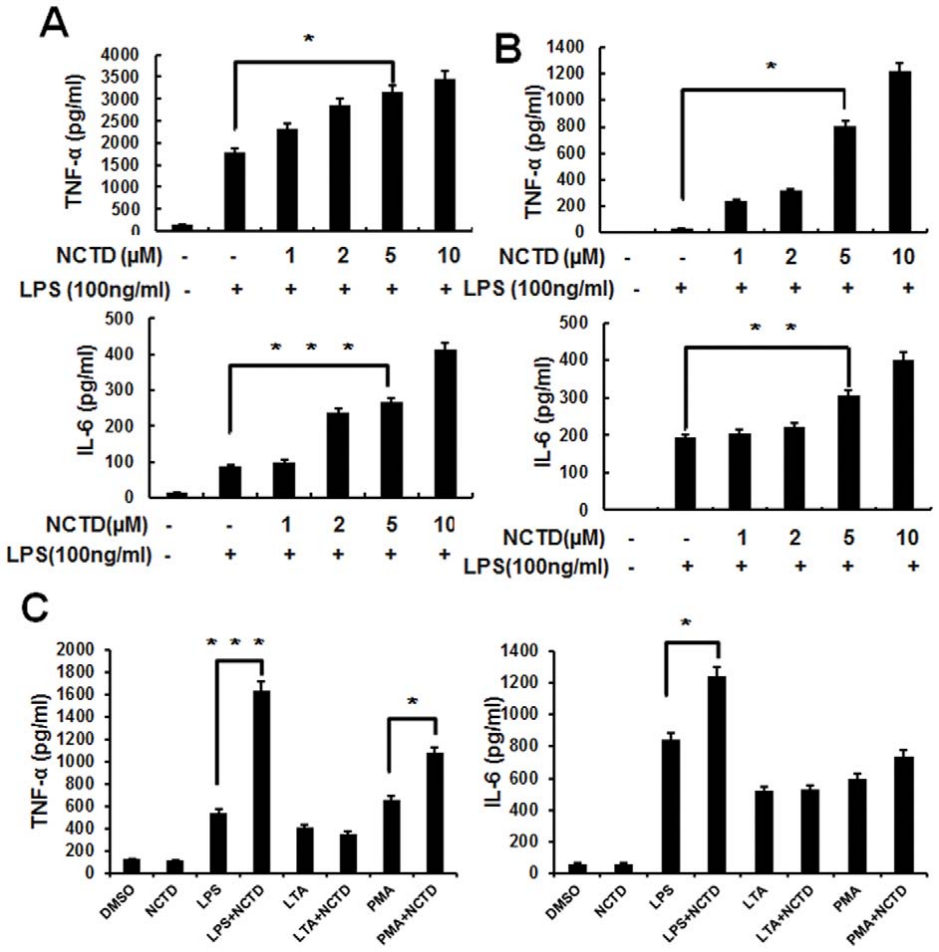
NCTD enhances production of pro-inflammatory cytokines in RAW264.7 and BMMs. A–B, RAW264.7 cells (A) and BMMs (B) were pretreated with PBS (containing 0.1% DMSO) or 1–10 μM NCTD for 24 h and activated with 100 ng/ml LPS for 6 h. The culture supernatants were then collected and analyzed by ELISA for cytokine production. C, Only LPS induced cytokine production was enhanced by NCTD obviously. The RAW264.7 cells were pretreated with 0.1% DMSO, 5 μM NCTD for 24 h before stimulation with LPS (100 ng/ml), PMA (100 nM) or LTA (10 μg/ml) for 6 h. The culture supernatants were then collected and analyzed by ELISA for cytokine production. Columns, means from three independent experiments with three duplicates; bars, SE (*, P<0.05; **, P<0.01 versus control).

### *In vivo* peritonitis mouse model

Peritonitis was induced as described previously with a little modification [22]–[24]. In brief, mice received an i.p injection of 1 ml vehicle (PBS) or NCTD (10 mg/kg) followed by an i.p injection with 8×10^7^ CFU of *E. coil 0111* in 1 ml PBS. The mice survivals were observed in the following 72 h. For bacterial visualization *in vivo*, *E. coli* BL21 were grown in Luria-Bertani broth (Difco) at 37°C and induced for GFP expression by IPTG at 16°C overnight. Bacteria were collected and washed by sterile PBS for bacteria number determination. The mice received an i.p injection of 1 ml vehicle (PBS), 100 μg/ml Ampicillin or NCTD (10 mg/kg) respectively followed by an i.p injection with 5×10^7^ CFU of E. coil-GFP (kanamycin resistance) in 1 ml PBS for 16 h. Then mice were killed and whole-body images were taken with the fluorescent stereomicroscope (Olympus America Inc, Melville, NY) coupled with a digital CCD camera (Olympus DP72). Animal procedures were approved by the institutional Animal Ethics Committee of East China Normal University.

**Figure 4:**
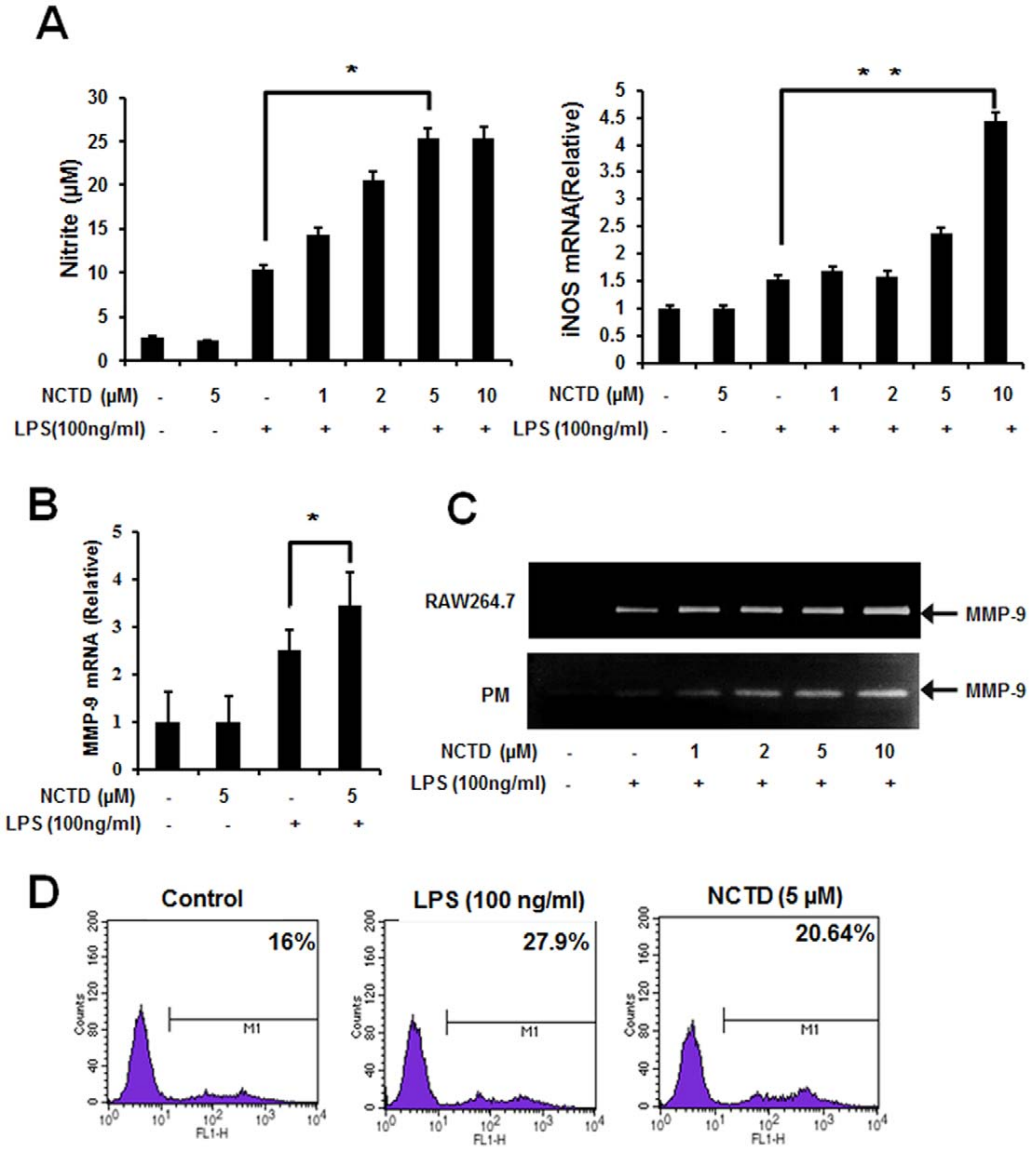
NO production, MMP-9 expression and phagocytosis was improved by NCTD significantly. A, NCTD increases LPS-induced NO production in macrophages. Macrophages were pretreated with PBS (containing 0.1% DMSO), 5 µM NCTD without stimulation or 1–10 µM NCTD stimulated with LPS (100 ng/ml) for 20 h, and then the production of nitric oxide was measured by Griess reaction (left). The mRNA levels of iNOS were assessed by Real time PCR (right). B, NCTD increases LPS-induced MMP-9 expression in mRNA level. RAW264.7 cells were pretreated with PBS (containing 0.1% DMSO) or 5 µM NCTD for 24 h and then stimulated with or without LPS (100 ng/ml) for 1 h. Total cellular RNAs were collected and subjected to Real time PCR analysis. C, NCTD enhances MMP-9 enzyme activity in RAW 264.7 and peritoneal macrophages. RAW264.7 cells and peritoneal macrophages were pretreated with PBS (containing 0.1% DMSO) or 1–10 µM NCTD for 12 h and then activated with LPS for another 12 h. The culture supernatants were then collected and MMP-9 levels were analyzed using gelatin zymography. D, NCTD facilitates phagocytosis of bacteria in macrophages. RAW 264.7 were pretreated with PBS (containing 0.1% DMSO), NCTD (5 µM) or NCTD with LPS (100 ng/ml) for 24 h, and then exposed to GFP-labeled *E.coli* for 30 min. The fluorescence were detected by flow cytometry after the cells were washed severely by ice cold PBS for three times. The numbers represent mean fluorescence intensity.

### Cell viability assay

RAW264.7 cells (2×10^4^ per well) were seeded into 96-well plate and treated with NCTD (5–100 μM) or CTD (1–10 μM) respectively for 24 h. Then the cell viability was determined by MTS method, following the manual of CellTiter 96 Aqueous One Solution Cell Proliferation assay (Promega) and the OD value was detected at 490 nm with VERSA max microplate reader (Molecular Devices).

**Figure 5:**
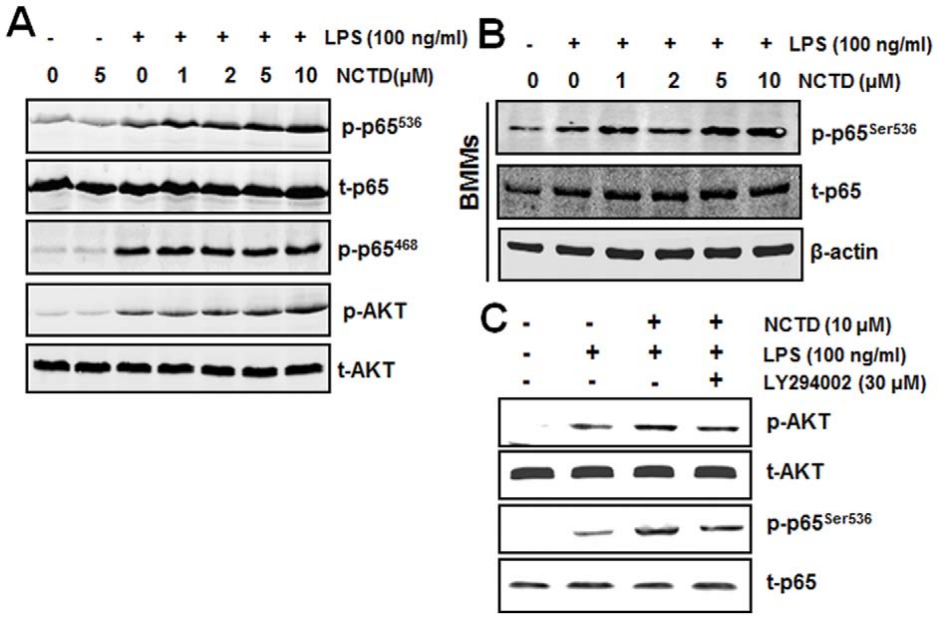
Regulation of NCTD on LPS induced p65 phosphorylation. A, p65 phosphorylation at Ser536 was up-regulated by NCTD in macrophages. RAW264.7 cells were treated with PBS (containing 0.1% DMSO), 5 µM NCTD without stimulation or 1–10 μM NCTD for 24 h and stimulated with 100 ng/ml LPS for different time. The levels of total proteins and their phosphorylated form in the cellular lysates were analyzed using Western blotting. B, NF-κB p65 phosphorylation was up-regulated by NCTD in BMMs. BMMs were treated by PBS (containing 0.1% DMSO) or 1–10 µM NCTD and stimulated with 100 ng/ml LPS for different time. Total cell lysates obtained at 15 min after the activation by LPS and the levels of total p65 and its phosphorylated form in the cellular lysates were analyzed using western blotting. C, PI3K inhibitor LY294002 blocks phosphorylation of AKT and p65. After pretreated with NCTD for 24 h, RAW264.7 macrophages were treated with 30 μM LY294002 for 40 min and then stimulated with 100 ng/ml LPS for the indicated time periods. The phosphorylation of AKT and p65 were detected by Western blotting. Three independent experiments were performed.

### Nitrite Quantification

RAW264.7 cells were treated with LPS (100 ng/ml) and NCTD (1–10 μM) in 96-well plates, NO_2_^−^ concentration in culture medium was measured to assess NO production. In brief, 50 μl of sample aliquots were mixed with same volume Griess reagent (1% sulfanilamide/0.1% naphthylethylene diamine dihydrochloride/2% phosphoric acid) in a 96-well plate and incubated at 25°C for 10 min. The absorbance at 550 nm was measured on a microplate reader. NaNO_2_ was used as a standard to calculate NO_2_^−^ concentrations.

**Figure 6:**
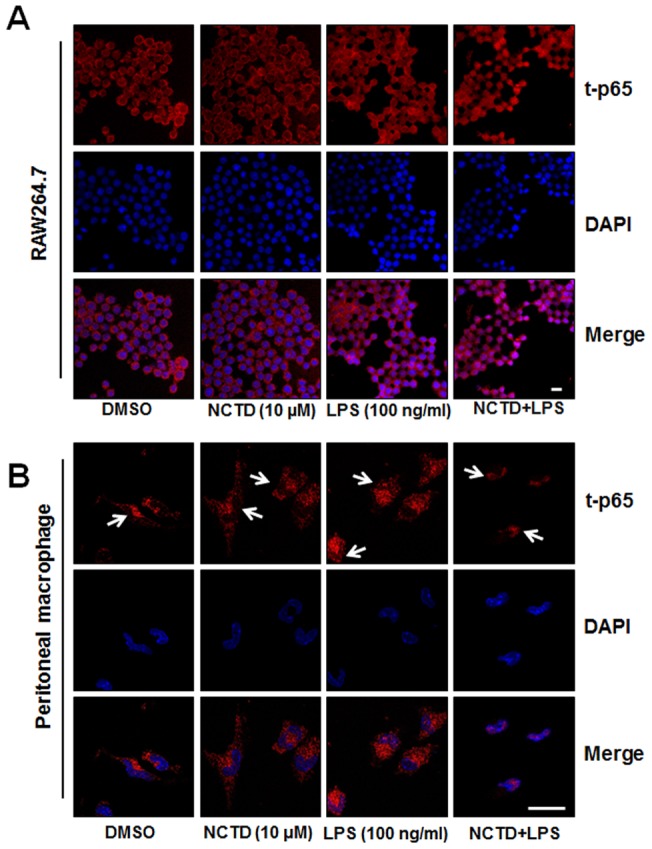
NCTD facilitates NF-κB nuclear translocation. A–B, RAW264.7 cells (A) and peritoneal macrophages (B) were stimulated with 100 ng/ml LPS in absence or presence of 5 µM NCTD that had been added 24 h before. When LPS addition was added for 15 min, subcellular location of NF-κB p65 subunit was detected using immunofluorescence assay. Scale bar, 10 μm. One of three experiments was shown.

### Phagocytosis Assay

RAW264.7 cells (1×10^6^ per well) were treated with or without LPS (100 ng/ml) and 10 μM NCTD for 24 h, and then 5×10^8^ CFU of E. coil-GFP were added for 30 min. Finally, free bacteria were washed by ice cold PBS three times to remove outside bacteria, and then the swallowed bacteria were detected by fluorescence-activated cell sorting (FACS) [25], [26]. The percentages of GFP cells population after phagocytosis were calculated according to blank control.

**Figure 7:**
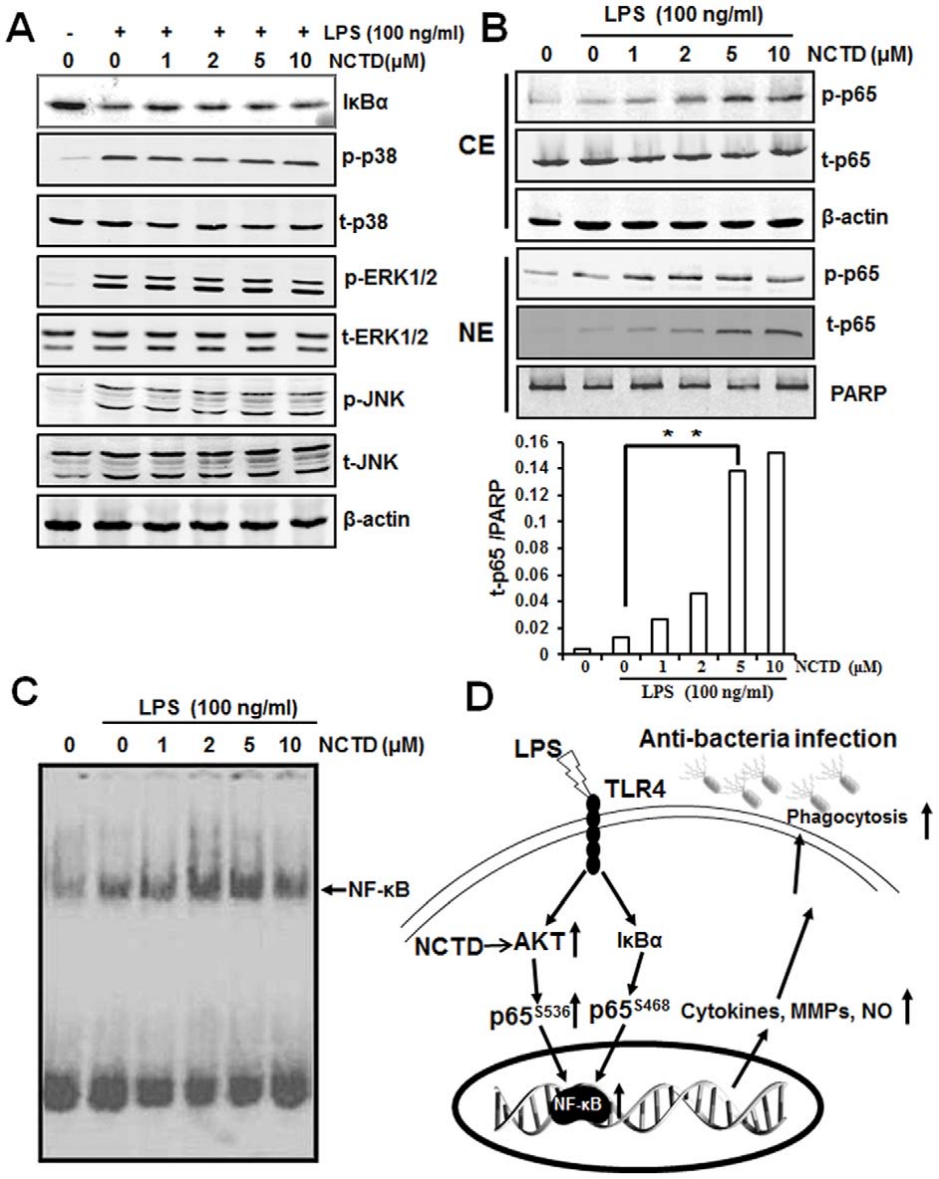
Regulation of NCTD on LPS induced NF-κB activation. A. Little influence was observed by NCTD on IκBα and MAPK signaling pathway. RAW264.7 cells were treated with PBS (containing 0.1% DMSO) or 1–10 μM NCTD for 24 h and then stimulated with 100 ng/ml LPS for different time. The protein levels of IκBα and MAPK signaling pathway in RAW264.7 cells were determined using western blotting. B. The translocation of p65 to nucleus was increased in NCTD treated macrophages. Cells (2×10^6^ cells/ml) were incubated with PBS (containing 0.1% DMSO) or 1–10 μM NCTD for 24 h and treated with 100 ng/ml LPS for 15 min. Cytoplasmic extracts (CE) and nuclear extract (NE) were prepared and analyzed by western blot analysis using specific antibodies. And the translocation of p65 to nuclear was also quantitatively analyzed. C. LPS induced DNA binding ability of NF-κB was enhanced by NCTD in a dose dependent manner. Cells were treated with PBS (containing 0.1% DMSO) or 1–10 μM NCTD at 37°C for 24 h and then activated with 100 ng/ml LPS for 30 min. Finally, nuclear extracts were prepared and then assayed for NF-κB activation by EMSA as described under Materials and Methods. Three independent experiments were performed. D, Diagram of NCTD facilitates LPS-mediated immune responses by up-regulation of AKT/NF-κB associated signaling pathway.

### ELISA Assay

For ELISA assay, RAW264.7 cells or murine BMMs (3×10^4^ cells/well) were seeded on 96-well-plate, and starved in DMEM containing 1% FBS overnight. Cells were treated with 1–10 μM NCTD for 24 h then stimulated with 100 ng/ml LPS for 6 h. Concentration of TNF-α and IL-6 in the cultured media was detected by ELISA kits according to the manufacturer's instructions (BD bioscience). The ELISA data representing mean values (±SD) were obtained in duplicate from at least three independent experiments.

### Gelatin zymography Assay

Firstly, RAW264.7 cells (2×10^4^ per well) were treated with or without LPS (100 ng/ml) and 1–10 μM NCTD for 12 h. Gelatinase activity was assayed by zymography analysis as previously described with some modifications [27]. Briefly, proteins in culture medium were normalized with Bradford reagent (Bio-Rad) and then separated by SDS/PAGE on a 10% (w/v) gel containing 1 mg/ml gelatin. The gel was washed twice in 2.5% (w/v) Triton X-100 solution and incubated overnight at 37°C in developing buffer (50 mmol/l Tris/HCl (pH 7.4), 10 mmol/l CaCl_2_, 5 mmol/l ZnCl_2_ and 0.05% Brij-35), stained with 0.5% Coomassie Blue and then destained in a 40% (v/v) methanol/10% (v/v) acetic acid solution. Proteolytic activity was evidenced as clear bands against the blue background of the stained gelatin.

### mRNA analysis

Total RNA was isolated from cultured RAW264.7 cells using TRIzol reagent (invitrogen) according to the manufacturer's instructions and Dnase I (RNase free) was added to avoid potential genomic DNA contamination. cDNA was then synthesized from isolated RNA by reverse transcription using the PrimeScript^TM^ 1st Strand cDNA Synthesis kit (TAKARA). Ten percent of the synthesized cDNA was used as a template for real-time PCR using the Taq DNA polymerase (TAKARA). Primers were designed to amplify mouse cytokines, chemokines and others genes are used. For IL-6 sense: 5′ TTC TTG GGA CTG ATG CTG 3′ and antisense: 5′ CTG GCT TTG TCT TTC TTG TT 3′; for TNF-α sense: 5′ TCC CTT TCA CTC ACT GGC 3′ and antisense: 5′ ACT TGG TGG TTT GCT ACG 3′; for IFN-β sense: 5′ TCC CTA TGG AGA TGA CGG 3′ and antisense: 5′ TCT GCT CGG ACC ACC AT 3′; for GM-CSF sense: 5′ TTT AGC GGC ACG AAC GA 3′ and antisense: 5′ GCC AGG ACT CAA GCG AAG 3′; for IL-1αsense: 5′ GCG AAT TCA CAG CAG TTG GAA TAA GCC GTG 3′ and antisense: 5′ CCA AGC TTG CAC AGA GTT GGA CAT GAC TGA AG 3′. PCR conditions were 94°C for 5 min followed by 30 cycles of 94°C for 30 s, 55°C for 30 s, 72°C for 30 s, and a final elongation at 72°C for 5 min. PCR products were run on the 1% agarose gel containing ethidium bromide.

### Western Blot Analysis

RAW264.7 cells were pretreated with or without 1–10 μM NCTD for 24 h and then stimulated with 100 ng ml^−1^ LPS for indicated time. The whole-cell extracts were prepared by RIPA buffer supplemented with different kinds of proteinase inhibitors. All samples were separated by SDS-PAGE to determine associated signaling, specific antibodies were used for different Western blot analyses, including p65/p-p65^ser536^/p-p65^ser468^, p38/p-p38, JNK/p-JNK, AKT/p-AKT, pThr202/Tyr204-ERK1/2 (extracellular signal-regulated kinase 1/2), and ERK1/2 (Cell Signaling Technology). To block PI3K/AKT signaling, RAW264.7 cells were incubated with LY294002 for 40 min at 37°C prior to stimulation with LPS (100 ng/ml) at 37°C as indicated.

### Immunofluorescence analysis

For Immunofluorescence assay, RAW264.7 cells (2×10^4^ per well) were seeded on coverslips in 24 well plate and treated with 10 μM NCTD for 24 h and treated with LPS for 15 min. Then cells were fixed with 4% paraformaldehyde in PBS for 15 min and permeabilized with 0.1% saponin in PBS for 15 min. After blocking with goat serum for 1 h, cells were incubated with Rabbit anti-phospho p65 antibody (Santa Cruz Biotechnique). After incubating for 2 h at room temperature, the slides were washed with PBS for three times. HRP-conjugated or Fluorescein-conjugated secondary antibody were added for 1 h, then DAPI was used for counterstaining of nuclei. Coverslips were finally mounted in mounting medium (sigma) and fluorescent images were taken under the Laser Confocal Microscope (Leica TCS SP5).

### Electrophoretic Mobility Shift Assays (EMSA)

Nuclear extraction and EMSA were performed as described [28]. RAW264.7 cells were incubated for 24 h with different concentrations of NCTD, and then treated with 100 ng/ml LPS for 30 min. After washed three times with ice-cold PBS, cells were incubated in the lysis buffer A (10 mM HEPES pH 8.0, 10 mM KCl, 0.1 mM EDTA, 0.1 mM EGTA, 1 mM DTT, 1 mM PMSF, 10 μg/ml Leupeptine, 10 μg/ml Pepstatine A, 10 μg/ml Aprotinin) followed by scraping and transferred to a new 1.5 ml tube, vortex gently, spin at 12000 rpm for less than 1 min in a standard bench-top microcentrifuger and the nuclear pellet was resuspended in lysis buffer B (5 mM HEPES pH 8.0, 8% glycerol, 2 mM MgCl2, 0.2 mM EDTA, 0.5 mM DTT, 0.5 mM PMSF, 10 μg/ml Leupeptine, 10 μg/ml Pepstatine A, 10 μg/ml Aprotinin). Then KCl was added to a final concentration of 500 mM and the sample was incubated for 1 h at 4°C with vigorous shaking. The mixture was centrifuged for 1 to 2 min at 12000 rpm and aliquots of the supernatant (nuclear extract) were immediately frozen at −80°C. Protein concentration was determined with Bradford method (BIO-RAD, Laboratories), and diluted to 2 mg/ml. 2 μg of Nuclear extracts were prepared and subjected to EMSA using a ^32^P-radiolabeled NF-κB oligonucleotide probe (5′-AGTTGAGGGGACTTTCCC AGGC-3′).

### Statistical analysis

The data are presented as mean ± SE, and statistical comparisons between groups were performed using one-way ANOVA followed by Student's t-test. A P value≤0.05 was considered statistically significant.

## Results

### NCTD protects the mouse from acute peritonitis

The structure of NCTD, 3a,4,5,6,7,7a-Hexahydro-4,7-epoxyisobenzofuran-1,3-dione, is shown in Fig .1A. To investigate the function of NCTD in regulating innate immunity, we set up an acute peritonitis mouse model and the survival was monitored every 3 hours in the next 72 h. As shown in Fig. 1B, almost all the mouse injected with *E.coli 0111* died without NCTD. In contrast, only one NCTD-treated mouse succumbed to the infection during the same period (Fig. 1B). To further explore the influence of NCTD on the immune response to the invade bacteria, we obtained blood from NCTD and PBS treated peritonitis mice to measure the serum concentrations of TNF-α and IL-6. As shown in Fig. 1C the serum concentrations of TNF-α and IL-6 in NCTD treated mouse was much higher than PBS treated group, indicating that the response to the bacteria in NCTD-treated mice was severely increased.

We also used the GFP-labeled bacteria to evaluate the clearance of invaded pathogen after being treated with NCTD. As shown in Fig. 1 D, the intensity of green fluorescence (left) was reduced obviously in both NCTD and ampicillin treated mice which was consistent with the number of bacterial (right), suggesting the distinct decrease of bacteria. To confirm this suppression of bacteria by NCTD is not through suppressing to proliferation, we cultured *E.coli 0111* with or without NCTD for 24 h. But no significant decrease of bacteria was found in NCTD-treated bacteria (Fig. 1 E ). Taken together, our results suggested a critical role of NCTD in regulation of immune response and pathogens clearance in bacteria-mediated peritonitis.

### NCTD activates LPS-induced cytokines production in macrophages

To elucidate the influence of NCTD on proliferation and viability to the immune cells, we treated RAW264.7 cells with NCTD or CTD for 24 h. Our data indicated that the viability of RAW264.7 cells was little reduced by NCTD at the concentration less than 10 μM, whereas much more cell suppression was found in CTD treated cells in the same concentration (Fig. 2A). To demonstrate the regulation of NCTD through activating cytokine gene expression, total cellular mRNA was extracted from RAW264.7 cells with or without NCTD treatment to perform the Real time PCR assay. NCTD was found increasing LPS induced TNF-α, IL-6, IL-1α, GM-CSF and IFN-β mRNA expression in a dose dependent manner (Fig. 2B). We also detected the secretion of cytokine by ELISA in RAW264.7 (Fig. 3A) and bone marrow–derived macrophages (Fig. 3B). Consistent with RT-PCR data, the protein level of TNF-α and IL-6 were also increased by NCTD in a dose dependent manner. Whereas, single NCTD treatment or synergized with PMA (12-myristate 13-acetate) and LTA (agonist to TLR2) can't potentiate cytokine production in Q-PCR and ELISA assay (Fig. 2C and Fig. 3C). Our data further confirmed the positive regulation of LPS induced cytokines production by NCTD in RAW264.7 cells and BMMs.

### NCTD enhances macrophage-mediated immune responses

The releasing of NO by macrophages is important for immune system to eliminate the invaded pathogens [29], [30]. As shown in Fig. 4A (left), LPS induced NO production was enhanced by NCTD in a dose dependent manner. Meanwhile, the mRNA level of iNOS was also elevated by NCTD in Real-time PCR assay (Fig. 4A right). Furthermore, the LPS-induced MMP-9 (matrix metalloproteinase 9) expression was promoted obviously by NCTD in RAW264.7 (Fig. 4B) which is consistent with enzyme activity in both RAW264.7 (upper lane) and peritoneal macrophages (lower lane) in gelatin zymography assay (Fig. 4C). As the key innate immune function of macrophage, we also evaluated the macrophage mediated pathogen phagocytosis. As shown in Fig. 4D, the phagocytosis of GFP-labeled bacteria by RAW264.7 cells were increased obviously (from 16% to 20.64%) by pretreating with 5 μM NCTD for 24 h, which was similar with LPS treated cells. Taken together, our current data further confirmed positive regulation of NCTD in macrophages mediated immune responses.

### NCTD enhances LPS-induced p65 phosphorylation and AKT activation

The LPS induced NF-κB and MAPKs (mitogen-activated protein kinases) associated signaling pathways are very important in TLR4-mediated immune responses [31]. Therefore, we examined some key signaling components of these two pathways. As we can see from Fig. 5A, only LPS-induced phosphorylation of AKT and p65 at Ser536 increased obviously in NCTD-pretreated RAW264.7 cells. Whereas, phosphorylation p65 at Ser468 which could cause IκBα degradation was not influenced. Similar results were also observed in BMMs (Fig. 5B).

Notably, AKT is also involved in NF-κB signaling [33]. To further confirm that NCTD facilitates NF-κB activation through AKT activation, we pretreated cells with PI3K/AKT inhibitor (LY294002) to rescue NCTD enhanced NF-κB activation. As shown in Fig. 5C, the NCTD-elevated phosphorylation of p65 and AKT were both suppressed by LY294002 obviously, indicated that NCTD facilitated NF-κB activation is AKT dependent. Together, these results demonstrated that the regulation of immune response by NCTD is mainly through potentiating AKT/NF-κB signaling pathway.

### NCTD facilitates LPS induced nuclear translocation of p65

Following the phosphorylation of p65, the free NF-κB dimers translocate into the nucleus and bind to specific sequence to regulate the downstream genes expression. So we labeled p65 with red fluorescence to track the influence of NCTD on NF-κB translocation. As shown in Fig. 6, p65 localized in nucleus was increased obviously by LPS which was further enhanced by NCTD both in RAW264.7 (Fig. 6A) and peritoneal macrophages (Fig. 6B). Meanwhile, we checked MAPKs associated signaling pathways by western blotting assay, but little influence was observed by NCTD in LPS-induced RAW264.7 cells (Fig. 7A). Besides, LPS induced IκBα degradation was little affected either (Fig. 7A), indicating that NCTD advanced NF-κB activation was not through IκBα degradation. Whereas, LPS induced phosphorylation of p65 in cytoplasmic pool and p65 nuclear accumulation were promoted by NCTD in a dose dependent manner (Fig. 7B) which is consistent with immunofluorescence assay (Fig. 6A&B). Our data suggested that the activation of p65 by NCTD is the key steps in regulating NF-κB translocation.

### NCTD enhanced DNA binding ability of NF-κB

To determine the effect of NCTD on NF-κB DNA binding ability, the nuclear extracts were prepared and analyzed by EMSA. As shown in Fig. 7C, the DNA binding ability of NF-κB was increased obviously by LPS and then enhanced by NCTD in a dose dependent manner. Taken together, our data demonstrated that NCTD can synergize LPS induced AKT/p65 phosphorylation and NF-κB transcriptional activity which partially explain the mechanism of NCTD in regulating innate immunity (Fig. 7D).

## Discussion

Drug reprofiling which developed new indications for existing drugs or biologics has become much more popular within pharmaceutical industry, providing a way of reducing the risks and costs in drug development, and possibly shortening the time to the market [32]. As the most famous and successful drug reprofiling case, Viagra (phosphodiesterase (PDE) type 5 inhibitor sildenafil citrate) which was originally intended treatment for hypertension and angina has been found to induce penile erections in trial participants and was then developed by Pfizer for this indication. In this paper, the traditional anti-tumor drug NCTD has been found as a positive regulator in TLR4 mediated anti-infection immunity. Meanwhile, the immune system also play a paradoxical roles role during cancer development [33]. Thus, it will be very interesting for us to explore the potential functions and mechanism of NCTD in regulation of immune system.

The immune system not only provides host defenses against invaded pathogens but also surveille malignant mutant cells all around the body. A robust immune system can recognize the mutated antigen on the cancer cells and awakes immune cells including cytotoxic T cells, natural killer cells, and macrophages to eliminate cancer cells. Thus the stronger immune system will do great efforts to rescue cancer patients from sufferings. Unfortunately, most immune cells are much more sensitive than cancer cells and could be restrained obviously by most anti-tumor drug for their severe cytotoxicity [34]. The reduced immunity makes patients more sensitive to infection, as well as other serious illness [35], [36]. Finding anti-tumor drugs which can also up-regulate the immune responses in patients will be a novel avenue of cancer treatment. Although NCTD has been widely used as an anti-tumor drug, the regulation of NCTD on immune system is little elucidated.

Bacterial peritonitis is a serious and fatal complication due to the sustain impairment to immune systems in patients with cancer. Different kinds of immune cells have been found involved in the host defense against infection, among them macrophages could be the most important cells which have series immune functions including phagocytosis, antigen presentation, and immunomodulation through cytokine and chemokine production [37]. Although over activated immune responses could be harmful and even fatal to the patient, the appropriate up-regulation of immunity in infectious diseases is also helpful to reduce infection-induced lethality. Thus, the activation of macrophages mediated immune function by different compounds and signaling could be helpful to strength immunity and protect host from invading pathogens. In this study, our findings not only reflect the protective effects of NCTD in an acute peritonitis mouse model, but also show that NCTD facilitates the clearance of invaded bacterial (Fig. 1). Furthermore, NCTD enhances the secretion of cytokines such as IL-6, TNF-α and IFN-β (Fig. 2 and 3), NO production, MMP-9 induction and phagocytosis (Fig. 4) in a dose dependent manner in macrophages. These findings provide the first and solid evidence that NCTD could serve as a positive regulator to the macrophage mediated innate immunity.

One of the primary physiological functions of NF-κB is regulation to the immune response, such as cytokine production, antigen presentation, pattern recognition and phagocytosis. Although LPS-induced immune response is mainly through NF-κB and AP-1 (activator protein 1) associated signaling pathway, only the phosphorylation of NF-κB p65 at Ser536 was promoted by NCTD (Fig. 5). Furthermore, NCTD also facilitates the translocation of p65 and therefore enhances NF-κB transcriptional activities in macrophages (Fig. 6 and 7). These results further strengthened the positive regulation of LPS mediated immune responses by NCTD in macrophages. As the up-stream signaling of p65, we also checked the protein level of IκBα by western blotting, whereas the degradation of IκBα was little influenced by NCTD under the same condition (Fig. 7A). Although the degradation of IκBα could initial the NF-κB signaling pathway, the phosphorylation of p65 at Ser536 is not associated with IκBα degradation [38]–[40]. Moreover, many studies have demonstrated PI3K/AKT signaling pathway can activate the nuclear translocation of NF-κB complexes as well as transactivation efficiency through phosphorylation of p65 at Ser536 [41], [42]. Previous work reveals that PI3K inhibitor LY294002 can repress phosphorylation of p65 and thus repress NF-κB transactivation, but have no effect on the IL-1 stimulated degradation of IκBα [43]. Therefore we treated the RAW264.7 cells with PI3K inhibitor LY294002 to block NCTD originated p65 activation. As shown in Fig. 5C, NCTD-elevated AKT and p65 phosphorylation was rescued to LPS treated level by LY294002, suggested the key role of AKT in regulation of immune response facilitated by NCTD.

In conclusion, our data demonstrated another function and mechanism of NCTD in modulating macrophages mediated immune responses through AKT/NF-κB signaling pathway. Therefore, our results implied the potential of NCTD to be a positive regulator in anti-infection immunity which reprofile the old drug with a new indication.

## References

[1] Janeway CA Jr, Medzhitov R (2002) Innate immune recognition. Annu Rev Immunol 20: 197–216. 11861602 10.1146/annurev.immunol.20.083001.084359

[2] Ulevitch RJ (2000) Molecular mechanisms of innate immunity. Immunologic Research 21: 49–54. 10852101 10.1385/IR:21:2-3:49

[3] Medzhitov R (2007) Recognition of microorganisms and activation of the immune response. Nature 449: 819–826. 17943118 10.1038/nature06246

[4] Medzhitov R, Janeway CA Jr (1997) Innate immunity: the virtues of a nonclonal system of recognition. Cell 91: 295–298. 9363937 10.1016/s0092-8674(00)80412-2

[5] Medzhitov R, Janeway C Jr (1997) Innate immunity: impact on the adaptive immune response. Current Opinion in Immunology 9: 4–9. 9039775 10.1016/s0952-7915(97)80152-5

[6] Fraser IP, Koziel H, Ezekowitz RA (1998) The serum mannose-binding protein and the macrophage mannose receptor are pattern recognition molecules that link innate and adaptive immunity. Semin Immunol 10: 363–372. 9799711 10.1006/smim.1998.0141

[7] Pearson AM (1996) Scavenger receptors in innate immunity. Current Opinion in Immunology 8: 20–28. 8729442 10.1016/s0952-7915(96)80100-2

[8] Beutler B, Jiang Z, Georgel P, Crozat K, Croker B, et al (2006) Genetic analysis of host resistance: Toll-like receptor signaling and immunity at large. Annu Rev Immunol 24: 353–389. 16551253 10.1146/annurev.immunol.24.021605.090552

[9] Jin MS, Lee JO (2008) Structures of the toll-like receptor family and its ligand complexes. Immunity 29: 182–191. 18701082 10.1016/j.immuni.2008.07.007

[10] Hirschfeld M, Weis JJ, Toshchakov V, Salkowski CA, Cody MJ, et al (2001) Signaling by toll-like receptor 2 and 4 agonists results in differential gene expression in murine macrophages. Infect Immun 69: 1477–1482. 11179315 10.1128/IAI.69.3.1477-1482.2001 PMC98044

[11] Hajjar AM, O'Mahony DS, Ozinsky A, Underhill DM, Aderem A, et al (2001) Cutting edge: functional interactions between toll-like receptor (TLR) 2 and TLR1 or TLR6 in response to phenol-soluble modulin. J Immunol 166: 15–19. 11123271 10.4049/jimmunol.166.1.15

[12] Okahara H, Yagita H, Miyake K, Okumura K (1994) Involvement of very late activation antigen 4 (VLA-4) and vascular cell adhesion molecule 1 (VCAM-1) in tumor necrosis factor alpha enhancement of experimental metastasis. Cancer Res 54: 3233–3236. 7515767

[13] Carrel JE, Eisner T (1974) Cantharidin: potent feeding deterrent to insects. Science 183: 755–757. 4856601 10.1126/science.183.4126.755

[14] Chang C, Zhu Y, Tang X, Tao W (2011) The anti-proliferative effects of norcantharidin on human HepG2 cells in cell culture. Mol Biol Rep 38: 163–169. 20333548 10.1007/s11033-010-0090-6

[15] Luan J, Duan H, Liu Q, Yagasaki K, Zhang G (2010) Inhibitory effects of norcantharidin against human lung cancer cell growth and migration. Cytotechnology 62: 349–355. 20087654 10.1007/s10616-009-9250-8 PMC2978303

[16] Chen YJ, Chang WM, Liu YW, Lee CY, Jang YH, et al (2009) A small-molecule metastasis inhibitor, norcantharidin, downregulates matrix metalloproteinase-9 expression by inhibiting Sp1 transcriptional activity in colorectal cancer cells. Chem Biol Interact 181: 440–446. 19616522 10.1016/j.cbi.2009.07.004

[17] Huang Y, Liu Q, Liu K, Yagasaki K, Zhang G (2009) Suppression of growth of highly-metastatic human breast cancer cells by norcantharidin and its mechanisms of action. Cytotechnology 59: 209. 19649717 10.1007/s10616-009-9221-0 PMC2774572

[18] Kok SH, Cheng SJ, Hong CY, Lee JJ, Lin SK, et al (2005) Norcantharidin-induced apoptosis in oral cancer cells is associated with an increase of proapoptotic to antiapoptotic protein ratio. Cancer Lett 217: 43–52. 15596295 10.1016/j.canlet.2004.07.045

[19] Chen YJ, Tsai YM, Kuo CD, Ku KL, Shie HS, et al (2009) Norcantharidin is a small-molecule synthetic compound with anti-angiogenesis effect. Life Sci 85: 642–651. 19765597 10.1016/j.lfs.2009.09.003

[20] Liu L, Sakai T, Sano N, Fukui K (2004) Nucling mediates apoptosis by inhibiting expression of galectin-3 through interference with nuclear factor kappaB signalling. Biochem J 380: 31–41. 14961764 10.1042/BJ20031300 PMC1224150

[21] Odeh M, Sabo E, Srugo I, Oliven A (2004) Serum levels of tumor necrosis factor-alpha correlate with severity of hepatic encephalopathy due to chronic liver failure. Liver Int 24: 110–116. 15078474 10.1111/j.1478-3231.2004.0894.x

[22] Celada A, Gray PW, Rinderknecht E, Schreiber RD (1984) Evidence for a gamma-interferon receptor that regulates macrophage tumoricidal activity. J Exp Med 160: 55–74. 6330272 10.1084/jem.160.1.55 PMC2187421

[23] Zhao M, Yang M, Baranov E, Wang X, Penman S, et al (2001) Spatial-temporal imaging of bacterial infection and antibiotic response in intact animals. Proc Natl Acad Sci U S A 98: 9814–9818. 11481427 10.1073/pnas.161275798 PMC55535

[24] Zhang Z, Wang Z, Ren H, Yue M, Huang K, et al (2011) P2Y(6) agonist uridine 5′-diphosphate promotes host defense against bacterial infection via monocyte chemoattractant protein-1-mediated monocytes/macrophages recruitment. J Immunol 186: 5376–5387. 21444765 10.4049/jimmunol.1002946

[25] Steinkamp JA, Wilson JS, Saunders GC, Stewart CC (1982) Phagocytosis: flow cytometric quantitation with fluorescent microspheres. Science 215: 64–66. 7053559 10.1126/science.7053559

[26] Doyle SE, O'Connell RM, Miranda GA, Vaidya SA, Chow EK, et al (2004) Toll-like receptors induce a phagocytic gene program through p38. J Exp Med 199: 81–90. 14699082 10.1084/jem.20031237 PMC1887723

[27] Xu P, Wang Y, Piao Y, Bai S, Xiao Z, et al (2001) Effects of matrix proteins on the expression of matrix metalloproteinase-2, −9, and −14 and tissue inhibitors of metalloproteinases in human cytotrophoblast cells during the first trimester. Biol Reprod 65: 240–246. 11420245 10.1095/biolreprod65.1.240

[28] Rosenberger CM, Finlay BB (2003) Phagocyte sabotage: disruption of macrophage signalling by bacterial pathogens. Nat Rev Mol Cell Biol 4: 385–396. 12728272 10.1038/nrm1104

[29] Willenborg DO, Staykova MA, Cowden WB (1999) Our shifting understanding of the role of nitric oxide in autoimmune encephalomyelitis: a review. J Neuroimmunol 100: 21–35. 10695712 10.1016/s0165-5728(99)00212-x

[30] Esteve PO, Chicoine E, Robledo O, Aoudjit F, Descoteaux A, et al (2002) Protein kinase C-zeta regulates transcription of the matrix metalloproteinase-9 gene induced by IL-1 and TNF-alpha in glioma cells via NF-kappa B. J Biol Chem. 277: 35150–35155. 10.1074/jbc.M108600200 12130632

[31] Kim JH, Jeong JH, Jeon ST, Kim H, Ock J, et al (2006) Decursin inhibits induction of inflammatory mediators by blocking nuclear factor-kappaB activation in macrophages. Mol Pharmacol 69: 1783–1790. 16510559 10.1124/mol.105.021048

[32] Tobinick EL (2009) The value of drug repositioning in the current pharmaceutical market. Drug News Perspect 22: 119–125. 19330170 10.1358/dnp.2009.22.2.1303818

[33] de Visser KE, Eichten A, Coussens LM (2006) Paradoxical roles of the immune system during cancer development. Nat Rev Cancer 6: 24–37. 16397525 10.1038/nrc1782

[34] Lambert LA, Mansfield PF (2007) Cytoreductive surgery and perioperative intraperitoneal chemotherapy for colorectal carcinomatosis: if at first you don't succeed. Ann Surg Oncol 14: 3037–3039. 17726635 10.1245/s10434-007-9548-8

[35] Picozzi VJ, Pohlman BL, Morrison VA, Lawless GD, Lee MW, et al.. (2001) Patterns of chemotherapy administration in patients with intermediate-grade non-Hodgkin's lymphoma. Oncology (Williston Park) 15: 1296–1306; discussion 1310–1291, 1314. 11702959

[36] Worthington HV, Clarkson JE, Khalid T, Meyer S, McCabe M (2010) Interventions for treating oral candidiasis for patients with cancer receiving treatment. Cochrane Database Syst Rev: CD001972. 10.1002/14651858.CD001972.pub4 PMC7063978 20614427

[37] Aderem A, Underhill DM (1999) Mechanisms of phagocytosis in macrophages. Annu Rev Immunol 17: 593–623. 10358769 10.1146/annurev.immunol.17.1.593

[38] Douillette A, Bibeau-Poirier A, Gravel SP, Clement JF, Chenard V, et al (2006) The proinflammatory actions of angiotensin II are dependent on p65 phosphorylation by the IkappaB kinase complex. J Biol Chem 281: 13275–13284. 16513650 10.1074/jbc.M512815200

[39] Buss H, Dorrie A, Schmitz ML, Hoffmann E, Resch K, et al (2004) Constitutive and interleukin-1-inducible phosphorylation of p65 NF-{kappa}B at serine 536 is mediated by multiple protein kinases including I{kappa}B kinase (IKK)-{alpha}, IKK{beta}, IKK{epsilon}, TRAF family member-associated (TANK)-binding kinase 1 (TBK1), and an unknown kinase and couples p65 to TATA-binding protein-associated factor II31-mediated interleukin-8 transcription. J Biol Chem 279: 55633–55643. 15489227 10.1074/jbc.M409825200

[40] Sasaki CY, Barberi TJ, Ghosh P, Longo DL (2005) Phosphorylation of RelA/p65 on serine 536 defines an I{kappa}B{alpha}-independent NF-{kappa}B pathway. J Biol Chem 280: 34538–34547. 16105840 10.1074/jbc.M504943200

[41] Madrid LV, Wang CY, Guttridge DC, Schottelius AJ, Baldwin AS Jr, et al (2000) Akt suppresses apoptosis by stimulating the transactivation potential of the RelA/p65 subunit of NF-kappaB. Mol Cell Biol 20: 1626–1638. 10669740 10.1128/mcb.20.5.1626-1638.2000 PMC85346

[42] Madrid LV, Mayo MW, Reuther JY, Baldwin AS Jr (2001) Akt stimulates the transactivation potential of the RelA/p65 Subunit of NF-kappa B through utilization of the Ikappa B kinase and activation of the mitogen-activated protein kinase p38. J Biol Chem 276: 18934–18940. 11259436 10.1074/jbc.M101103200

[43] Sizemore N, Leung S, Stark GR (1999) Activation of phosphatidylinositol 3-kinase in response to interleukin-1 leads to phosphorylation and activation of the NF-kappaB p65/RelA subunit. Mol Cell Biol 19: 4798–4805. 10373529 10.1128/mcb.19.7.4798 PMC84278

